# Comparative proteomic analysis provides novel insights into the regulation mechanism underlying papaya (*Carica papaya* L.) exocarp during fruit ripening process

**DOI:** 10.1186/s12870-019-1845-4

**Published:** 2019-06-06

**Authors:** Bian Jiang, Siyan Ou, Ling Xu, Wanyi Mai, Meijun Ye, Haiping Gu, Tao Zhang, Changchun Yuan, Chenjia Shen, Jinxiang Wang, Kaidong Liu

**Affiliations:** 1 Life Science and Technology School, Lingnan Normal University, Zhanjiang, 524048 China; 2 Root Biology Center, College of Natural Resources and Environment, South China Agricultural University, Guangzhou, 510642 China; 3 College of Life and Environmental Sciences, Hangzhou Normal University, Hangzhou, 310036 China

**Keywords:** Differential expressed protein, Fatty acid metabolism, Fruit, Hormones, Papaya, Ripening

## Abstract

**Background:**

Papaya (*Carica papaya* L.) is a popular climacteric fruit, undergoing various physico-chemical changes during ripening. Although papaya is widely cultivated and consumed, few studies on the changes in metabolism during its ripening process at the proteasome level have been performed. Using a newly developed TMT-LCMS analysis, proteomes of papaya fruit at different ripening stages were investigated.

**Results:**

In total, 3220 proteins were identified, of which 2818 proteins were quantified. The differential accumulated proteins (DAPs) exhibited various biological functions and diverse subcellular localizations. The KEGG enrichment analysis showed that various metabolic pathways were significantly altered, particularly in flavonoid and fatty acid metabolisms. The up-regulation of several flavonoid biosynthesis-related proteins may provide more raw materials for pigment biosynthesis, accelerating the color variation of papaya fruit. Variations in the fatty acid metabolism- and cell wall degradation-related proteins were investigated during the ripening process. Furthermore, the contents of several important fatty acids were determined, and increased unsaturated fatty acids may be associated with papaya fruit volatile formation.

**Conclusions:**

Our data may give an intrinsic explanation of the variations in metabolism during the ripening process of papaya fruit.

**Electronic supplementary material:**

The online version of this article (10.1186/s12870-019-1845-4) contains supplementary material, which is available to authorized users.

## Background

Fruit is a mature ovary including carpel tissues, and several fleshy fruits are important components of human and animal diets [1]. Fruit ripening is a complex process defined by dramatic metabolisms and textural transformations, color changes, softening, nutritional ingredient accumulation and flavor compound production [1, 2]. Although softening increases the nutritional and flavor properties of fruit, it also leads to biomass loss and postharvest deterioration [3].

Fruit softening is a deep-studied process that involves cell wall degradation [4]. The cell walls of fruit are composed of pectins, poly-galacturonans, celluloses and hemicelluloses, and degradation of these components is the major cause of tissue softening during ripening [5]. The pectin matrix, composed of both linear and branched polysaccharides, mainly accumulates in the primary cell wall. Several specific enzymes, such as pectate lyase, polygalacturonase and pectin methylesterase, show essential roles in the hydrolysis of polysaccharide chains of the pectin matrix [5, 6].

For climacteric fruits, the softening phenomenon is also controlled by a complex series of non-enzymatic events [7, 8]. During fruit ripening, abundant flavor and nutritional metabolites significantly change, including sugars, organic acids, lipids and amino acids, leading to modification of fruit texture [9]. For instance, sugars, the major flavor and aroma components, increase rapidly apricot fruit during development and ripening process [10]. In starch-rich fruit, starch and organic acids are converted to sugar by a metabolic degradation pathway [11]. Additionally, lipid components of fruits are assumed to enhance the formation of aromas and flavors in most climacteric fruits [12]. The variations in the composition of fatty acids during the fruit ripening process have been revealed in different climacteric fruits [13]. For example, most fatty acids increased during the ripening of mango fruit [14].

Papaya (*Carica papaya* L.), a climacteric fruit widely planted and consumed, displays quick softening and a short shelf-life [15, 16]. Once the climacteric fruit is mature, its soft texture and susceptibility to pathogenic fungi make the storage life short [17]. To overcome the commercial trading problem and prolong the shelf life, many studies on papaya have been carried out [18]. In papaya fruit, the physico-chemical changes during ripening are affected by the expression of ripening-related genes. A previous transcriptome identified 414 ripening-related genes, including those of MADS-box, NAC and ERF families, providing molecular information on papaya [19]. Several years ago, a fruit-specific expressed subtilase gene was identified in papaya by rapid amplification of cDNA ends and PCR primer walking techniques [20]. Application of 1-MCP (1-methylcyclopropene) can block ethylene signaling and so improve the shelf life [21]. Expression analysis of ethylene signaling pathway-related genes showed a relationship between chilling injury and ethylene signaling in papaya [16]. Physiological degradation of pectin in papaya cell walls has also been studied. For example, up-regulated expression levels of genes for PG, endoxylanase and β-galactosidase (β-GAL) were positively correlated with postharvest papaya fruit ripening [22, 23]. However, molecular and enzymatic mechanisms underlying the softening of papaya fruit during ripening remain largely unknown.

Many studies on the physiological and genomic variations during the ripening process of papaya fruit have been done [19, 23, 24]. To date, several proteomic data sets of papaya have been published. Using the 2-DE approach, differential accumulated proteins responsive to 1-MCP treatment during ripening have been identified [25]. Another comparative proteomic analysis showed that certain proteins, such as enolase, esterase and ADH3, show an essential role in maturation of somatic papaya embryo cells [26]. Recently, a differential proteome of virus-infected pre-flowering *C. papaya* vs. uninfected plants was published [27]. In our study, we employed an integration of the basic HPLC fractionation and LC–MS/MS approach to identify a total of 2818 differential accumulated proteins (DAPs) of papaya fruit during ripening. Our results offer important information about how to expend the shelf life of postharvest papaya fruit.

## Methods

### Plant materials and sampling

Two-year-old papaya trees of cultivar ‘Sunrise’ were planted in a 4 m × 4 m arrangement. The seedlings of papaya tree were purchased by the Yixin horticulture company (Zhanjiang, China), who provided permission to use the seedlings for our scientific research. The experimental station was at Lingnan Normal University campus in Zhanjiang, China. Plant experiment was performed in the Life Science and Technology School, Lingnan Normal University, according to a plant protocol approved by the Research Ethics Committee of Lingnan Normal University. All papaya trees were fertilized twice a week with a standard nutrient solution_._ Two fruit groups at similar color break stage (5% ≤ peel color ≤10% yellow) were harvested. The fruit coloring index was defined and calculated as follows: $\mathrm{coloring}\ \mathrm{index}=\sum \frac{\left(\mathrm{coloring}\ \mathrm{grade}\times \mathrm{number}\ \mathrm{of}\ \mathrm{fruit}\right)}{\mathrm{total}\ \mathrm{number}\ \mathrm{of}\ \mathrm{fruit}}.$

There are three papaya in one sample, and three samples for one group. The selected fruit was cleaned with deionized H_2_O, and then dipped into 0.2% of hypochloride solution for 15 min to eliminate potential microbes in growth chamber of our lab. The conditions of the chamber were set as: temperature of 26 ± 1 °C, a light/dark cycle of 16/8 h and humidity of 65–70%.

### Physiological parameter measurements

Measurements of the physiological parameters of papaya fruit were performed according to the previous study [15]. Three biological replicate were used. Briefly, the firmness of fruit was determined using a GY-J mini fruit tester (Top Instrument Co Ltd., Shanghai, China). For total soluble solid (TSS) measurement, 5.0 g of fruit pulp from three independent fruit was crushed manually with a mortar and pestle to extract the juice. The TSS (%) was measures using a J1-3A refractometer (Guangdong Scientific Instruments, Guangzhou, China). The same juice was titrated against 0.1 N NaOH until a faint pink color was obtained at the end point and titratable acidity was expressed as percentage citric acid. For respiration rate determination, three independent papaya fruit from each group were weighed and sealed in 2 L containers at 25 °C. The concentration of CO_2_ in each container was measured using an infrared Li-6262 CO_2_/H_2_O gas analyzer (LI-COR, USA).

### Observation by light microscopy and scanning electron microscopy

Fragments (0.5 cm long) of surface tissues were collected from whole fruit at different ripening stages. Samples were fixed in formalin acetic acid (FAA)_50_ for 48 h and transferred into 70% ethanol. The tissue samples were dehydrated in several varied concentrations of ethanol, and then embedded in wax. Next, fruit samples were sliced into a number of 12-μm thick sections using a RM2255 rotary microtome (Leica, Wetzlar, Germany) and spreaded on glass slides. The wax sections were eliminated in xylol:ethanol (1:1, v/v) solution for 5 min and rehydrated in several decreasing concentrations of ethanol, including 100, 95, 85 and 70%, for 1 min each concentration. The sample slides were stained in 3% safranin for 24 h and washed with a series of increasing ethanol concentrations of 70, 85 and 95%, for 5 s each concentration. Next, the sample slides were dipped into 1% fast green dye solution for 3 s each, treated twice with 100% of ethanol for 5 s each, and transferred into xylol:ethanol (1:1, v/v) solution for 5 min. All slides were mounted with synthetic resin and observed under a LSM510 laser scanning system (Carl Zeiss, Wetzlar, Germany).

Samples for scanning electron microscopy were prepared as above. Sample segments were gently dipped into xylol at 45 °C for 4 h to clean the wax. The samples were treated with xylol:ethanol (1:1, v/v) solution and 100% ethanol for 1 h and then put in tert-butyl alcohol. Finally, dried segments were metalized with gold for scanning electron microscope observation.

### Protein extraction

Protein extraction was performed according to the previous study [28]. Approximately 0.5-g exocarp samples were kept in N_2_ and pulverized. The fine powder was suspended with pre-cooled lysis buffer, containing 8 M urea, 2 mM EDTA, 10 mM DTT and 1% Protease Inhibitor Cocktail, on ice overnight. The precipitate was removed at 15,000 *g* by centrifugation at 4 °C for 15 min. The final supernatants were redissolved in buffer containing 8 M urea, and 100 mM triethylammonium bicarbonate (TEAB) and quantified by a 2-D Quant kit (GE Healthcare, Beijing, China).

### Digestion and tandem mass tag (TMT) labeling

The soluble samples were dissolved in 10 mM dithiothreitol buffer for 1 h at room temperature. The sample solution was alkylated with 20 mM iodoacetamide for 30 min at 25 °C in darkness. Then, the samples were redissolved in 100 μL of TEAB (100 mM). For the first digestion, trypsin (Sequencing Grade Modified Trypsin, V5113, Promega) was added at 1:50 (trypsin:protein) overnight and 1:100 (trypsin:protein) for a second digestion for 4 h. Approximately 200 μg of protein for each sample was treated for further experiments. The digestion was carried out at 25 °C.

Then, samples were desalted by a Strata X 33 μm C18 SPE column (8B-S100-AAK, Phenomenex, Torrance, CA, USA) and reconstituted using a 6-plex TMT kit (Thermo-Scientific, Rockford, USA). The supernatants were collected by centrifugation at 10,000×*g* for 15 min. Finally, the supernatants were subsequently labeled randomly with different TMT agents (Thermo-Scientific). Specifically, the amount of TMT-label solution needed to label 100 μg protein sample was reconstituted in 24 μL of acetonitrile. The mixture was incubated at room temperature for 2 h. At last, samples were pooled and dried b for further analysis.

### Fractionation and LC–MS/MS analysis

The peptide samples were separated into various fractions by reverse-phase HPLC using an Agilent 300Extend C_18_ column (5 μm particles, 4.6 mm ID, 250 mm length, Santa Clara, CA, USA). The samples were detected at wavelength 250 nm. Then, the peptides were combined into 18 fractions and dried.

Fractionated samples were dissolved in 0.1% formic acid (FA) solution and directly loaded onto a Acclaim PepMap 100 reversed-phase pre-column (25 cm length, 2 μm, Thermo, Shanghai, China). The gradient comprised increases in solution B, containing 0.1% FA in 98% ACN, from 4 to 22% in 26 min, from 22 to 35% in 8 min, climbing to 80% in 3 min and maintained at 80% for 3 min, with a constant flow rate of 320 nL/min using an EASY-nLC 1000 UPLC system (Thermo, Shanghai, China). The peptide samples were subjected to an Nano-Spray Ionization source followed by MS/MS in a Q Exactive™ (Thermo, Shanghai, China) coupled to the online UPLC [29]. The MS proteomics data have been set to the ProteomeXchange Consortium by PRIDE partner repository program under identifier PXD008871.

### Validation of DAPs by parallel reaction monitoring (PRM)

The PRM method was used to confirm the changes in DAPs identified in the LC–MS–TMT analysis. In this experiment, three independent samples were identified by an acquired MS/MS spectrum. Three biological replicates were used. The tryptic peptides were dissolved in solution (0.1% FA), directly loaded onto a reversed-phase analytical column (150 mm length, 75 μm, Thermo, Shanghai, China). The gradient of solution B containing 0.1% FA and 98% CAN, which comprised an increasing contents of 6 to 25% during 38 min, from 25 to 38% in 14 min, climbing to 80% in 4 min, and maintained at 80% for 4 min, with a constant flow rate of 400 nL/min.

### Database search

The MS/MS data were processed using MaxQuant with integrated Andromeda search engine v.1.5.2.8. MS/MS data were searched against the papaya genome data (http://phytozome.jgi.doe.gov/pz/portal.html) concatenated with a reverse decoy database. Trypsin/P was used as cleavage enzyme allowing up to two missing cleavages. For precursor ions, the mass error was set to 10 ppm, and for fragment ions the mass error was set to 0.02 Da. For protein quantification, TMT 6-plex was selected in MaxQuant. For peptide and protein identification, the false discovery rate (FDR) threshold was adjusted to < 1% and the peptide ion score was set to ≥20.

### Annotation methods

Gene ontology (GO) annotation of our proteome was derived from the UniProt-GOA database (www. http://www.ebi.ac.uk/GOA/). Firstly, all identified protein IDs were converted to UniProt IDs and mapped to GO IDs. Then, InterProScan soft was used to annotate the proteins that were not annotated by UniProt-GOA database based on protein sequence alignment method.

Kyoto Encyclopedia of Genes and Genomes (KEGG) database (http://www.genome.jp/kegg/) was used to annotate protein pathways. Firstly, KEGG online service tools KAAS (http://www.genome.jp/tools/kaas/) was used to annotate the KEGG description of proteins. Then, the annotation results were mapped to the KEGG pathway database using online tool KEGG Mapper (http://www.kegg.jp/kegg/mapper.html).

A subcellular localization predication software, WOLFPSORT, was used to predict subcellular localization (https://wolfpsort.hgc.jp/).

### Functional enrichment analysis

Within the DAPs, a Fisher’s test was used to analyze the GO and KEGG functional enrichments. Correction hypothesis was carried out using the FDR control method. GO and KEGG categories with a corrected *P*-value < 0.05 were considered significant. K-means cluster was analyzed using the MeV software. In order to meet the requirements of the hierarchical clustering method [30].

### Fatty acid content determination

Fatty acid methyl ester was prepared according to the previous study [31] with several minor alterations. Three biological replicates were used. A 40-mg fruit sample was put into a 15-mL tube. Then, 2 ml of mixture of ethyl ether and n-hexane (1:1, v/v), 2 ml of 2-mol/l KOH in methanol solution and 2 ml of methanol were added to the glass tube. The mixture solution was kept in a water bath at 50 °C for 30 min with shaking. When the solution was cool, 2 mL of deionized water was added to the tube. The up layer was collected and loaded to GC–MS. A constant flow rate of 0.8 mL/min was used as the carrier gas. The temperature of oven was set initially at 170 °C for 30 s, then raised to 230 °C at a rate of 5 °C/min and kept at 230 °C for 13 min. The MS was applied in an electron mode at 70 eV with a scan range of 30–450 m/z.

## Results

### Micromorphology and physiology changes of papaya fruit during ripening

**Fig. 1:**
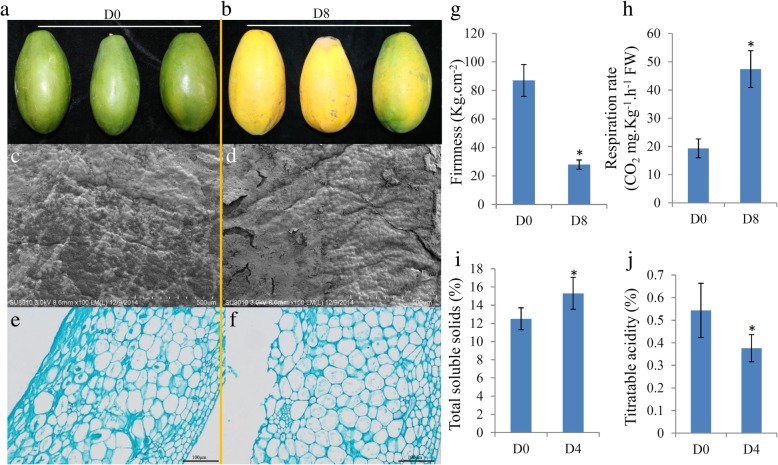
Micromorphology and physiology changes of papaya fruit during ripening process. Pictures of papaya fruit under time points D0 (**a**) and D8 (**b**) after harvest. Light microscopy observation of surface tissues collected from the fruit under time points D0 (**c**) and D8 (**d**) after harvest. Scanning electron microcopy observation of surface tissues collected from the fruit under time points D0 (**e**) and D8 (**f**) after harvest. The physiological features, including firmness (**g**), respiration rate (**h**), total soluble solids (**i**) and titratable acidity (**j**), of papaya fruit under time points after harvest

Two groups of papaya fruit (five per group) at the time points of 0 and 8 d after harvest were collected and analyzed. Papaya peel during ripening exhibited morphological changes in the epidermal and sub-epidermal cells. Fruit at 0 d had a glossy surface and at 8 d had an irregular surface (Fig. 1a–d). On 0 day, peel was comprised of epidermal cells with bulliform shape (Fig. 1e). By day 8, the upper layers showed horizontal elongation with an associated decrease in cell height (Fig. 1f). The firmness of fruit was rapidly down-regulated during ripening. Respiration rate of fruit was much higher in 8 d than that in 0 d. The papaya had a significantly higher TSS and lower TA during fruit ripening (Fig. 1g–i).

### Quality control and quantitative proteome analysis

**Fig. 2:**
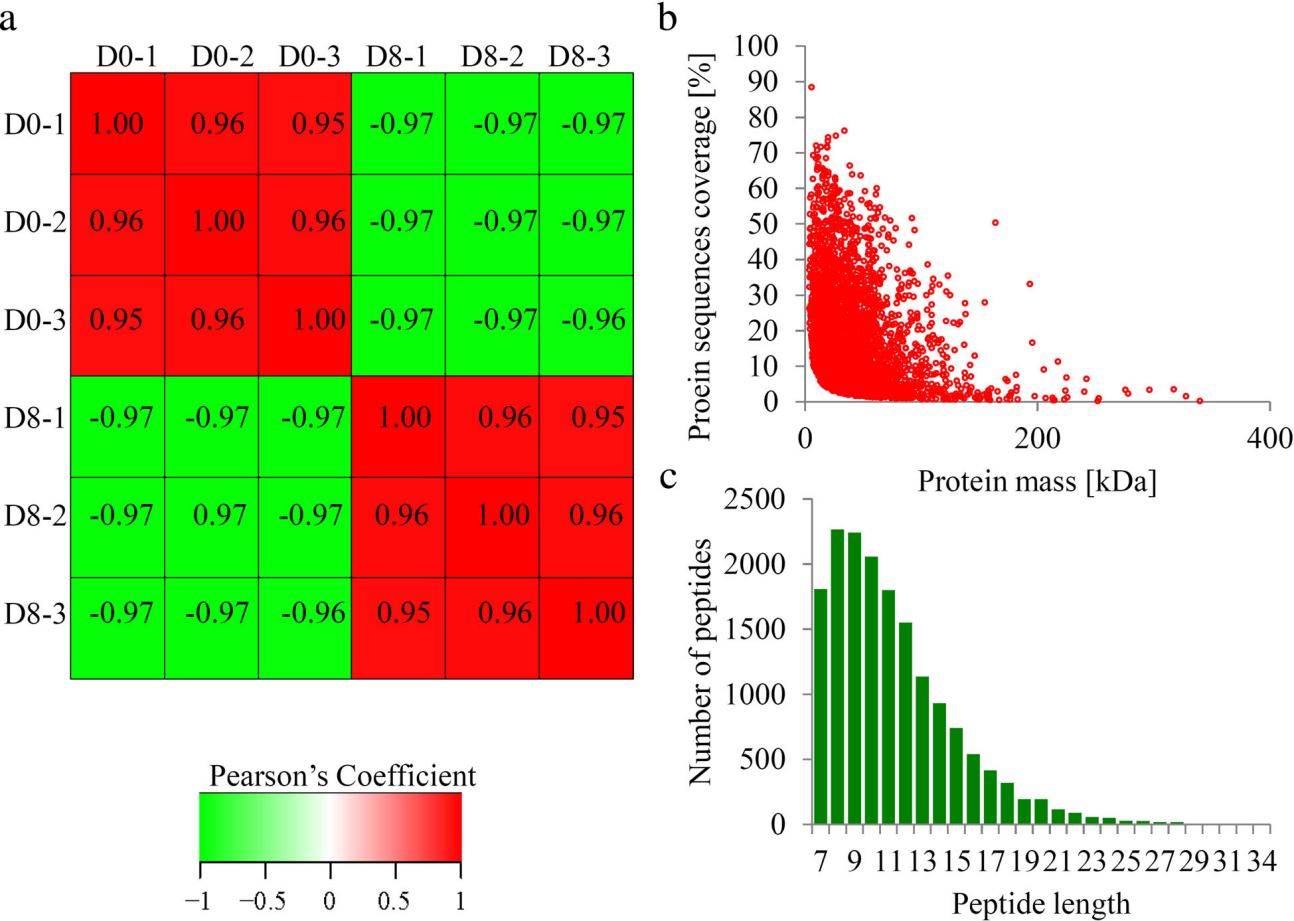
Quantitative proteome analysis and QC validation of MS data. Protein were extracted in three biological replicates for each fruit group. Proteins were trypsin digested and analyzed by HPLC-MS/MS. (**a**) Pearson’s correlation of protein quantitation. (**b**) Mass delta of all identified peptides. (**c**) Length distribution of all identified peptides

The changes in the proteome of papaya fruit at two different ripening stages were quantified. The correlation coefficients of two stages × three replicates indicated a good repeatability of MS data (Fig. 2a). The mass errors of the identified peptides were measured – most mass errors were < 0.02 Da, and their distribution was near zero (Fig. 2b). The lengths of all identified peptides were within 8–16 Aa (Fig. 2c). To get more detailed information on the identified and quantified proteins, GO, KEGG, domain and subcellular localization annotations of all proteins were showed in Additional file 1**.**

### Identification of DAPs during fruit ripening

A total of 960 DAPs, including 425 up- and 535 down-regulated proteins, were identified (Additional files 2 and 3). Among the DAPs, the top five up-regulated proteins were a triphosphate hydrolase superfamily protein (9.3 fold), followed by an inhibitor of trypsin and hageman factor-like protein (8.4 fold), a groES-like zinc-binding dehydrogenase family protein (7.7 fold), an 1-aminocyclopropane-1-carboxylate oxidase (7.6 fold) and a glutamine aminotransferase (7.6 fold). The top five down-regulated proteins were three chlorophyll a–b binding proteins (34.5, 21.2 and 14.7 fold, respectively), a PsaB protein (16.7 fold) and a Photosystem I 11 K family protein (15.6 fold) (Additional file 3). Subcellular locations of the DAPs were predicted (Additional file 4). For the induced proteins, a total of 11 different groups were identified, such as chloroplast- (146 proteins), cytosol- (122), nucleus- (58), mitochondria- (29) and plasma membrane-localized proteins (28 proteins) (Additional file 2). For reduced proteins, 14 components were identified, including chloroplast- (193 proteins), cytosol- (167), nucleus- (58), extracellular- (25) and plasma membrane-localized protein (25) (Additional file 2).

### Enrichment analysis of DAPs during fruit ripening

**Fig. 3:**
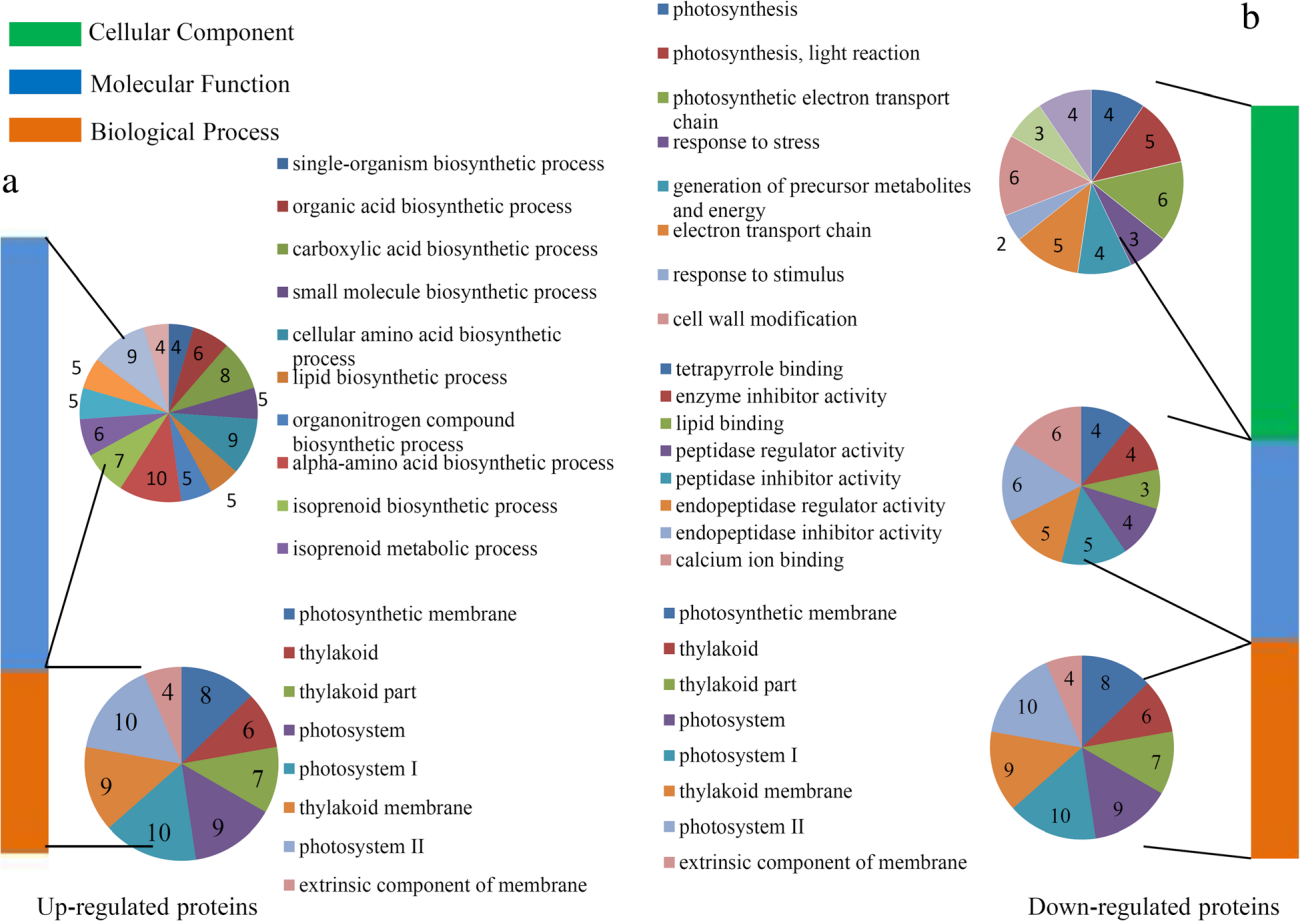
GO enrichment analysis of DAPs. (**a**) Distribution of the up-regulated proteins with GO annotation. Different color blocks represent different terms, including cellular component, molecular function, and biological process. Number of the up-regulated proteins in each second level term was showed in a pie chart. (**b**) Distribution of the down-regulated proteins with GO annotation. Different color blocks represent different terms, including cellular component, molecular function, and biological process. Number of the down-regulated proteins in each second level term was showed in a pie chart

In total, 332 DAPs were assigned to at least one GO term. For up-regulated proteins, the highly enriched ‘Molecular Function’ GO terms were ‘alpha-amino acid biosynthetic process’, ‘cellular amino acid biosynthetic process’, ‘carboxylic acid biosynthetic process’, ‘pigment metabolic process’, ‘cellular lipid metabolic process’, ‘isoprenoid metabolic process’, ‘organic acid biosynthetic process’ and ‘small molecule biosynthetic process’; and the significantly enriched ‘Biological Process’ GO terms were ‘Photosystem I’, ‘Photosystem II’, ‘thylakoid membrane’, ‘extrinsic component of membrane’, ‘photosynthetic membrane’ and ‘thylakoid part’ (Fig. 3a). For down-regulated proteins, the highly enriched ‘Biological Process’ GO terms were related to ‘Photosystem I’, ‘Photosystem II’ and ‘thylakoid membrane’; within the ‘Molecular Function’, the most significantly enriched terms were ‘calcium ion binding’, ‘endopeptidase inhibitor activity’ and ‘endopeptidase regulator activity’; and the most enriched terms in the ‘Cellular Component’ were ‘cell wall modification’, ‘photosynthetic electron transport chain’ and ‘photosynthesis, light reaction’ (Fig. 3b).

The up-regulated proteins were mostly associated with ‘Biosynthesis of secondary metabolites’ and ‘Fatty acid biosynthesis’; and the down-regulated proteins were mostly involved in ‘Photosynthesis’ and ‘Glyoxylate and dicarboxylate metabolism’ (Additional file 5).

The up-regulated proteins mostly contained a ‘Isopenicillin N synthase-like’ domain and down-regulated proteins mostly contained a ‘Chlorophyll a/b binding protein’ domain (Additional file 6).

### Involvement of pathogen defense-related proteins in the fruit ripening process

**Fig. 4:**
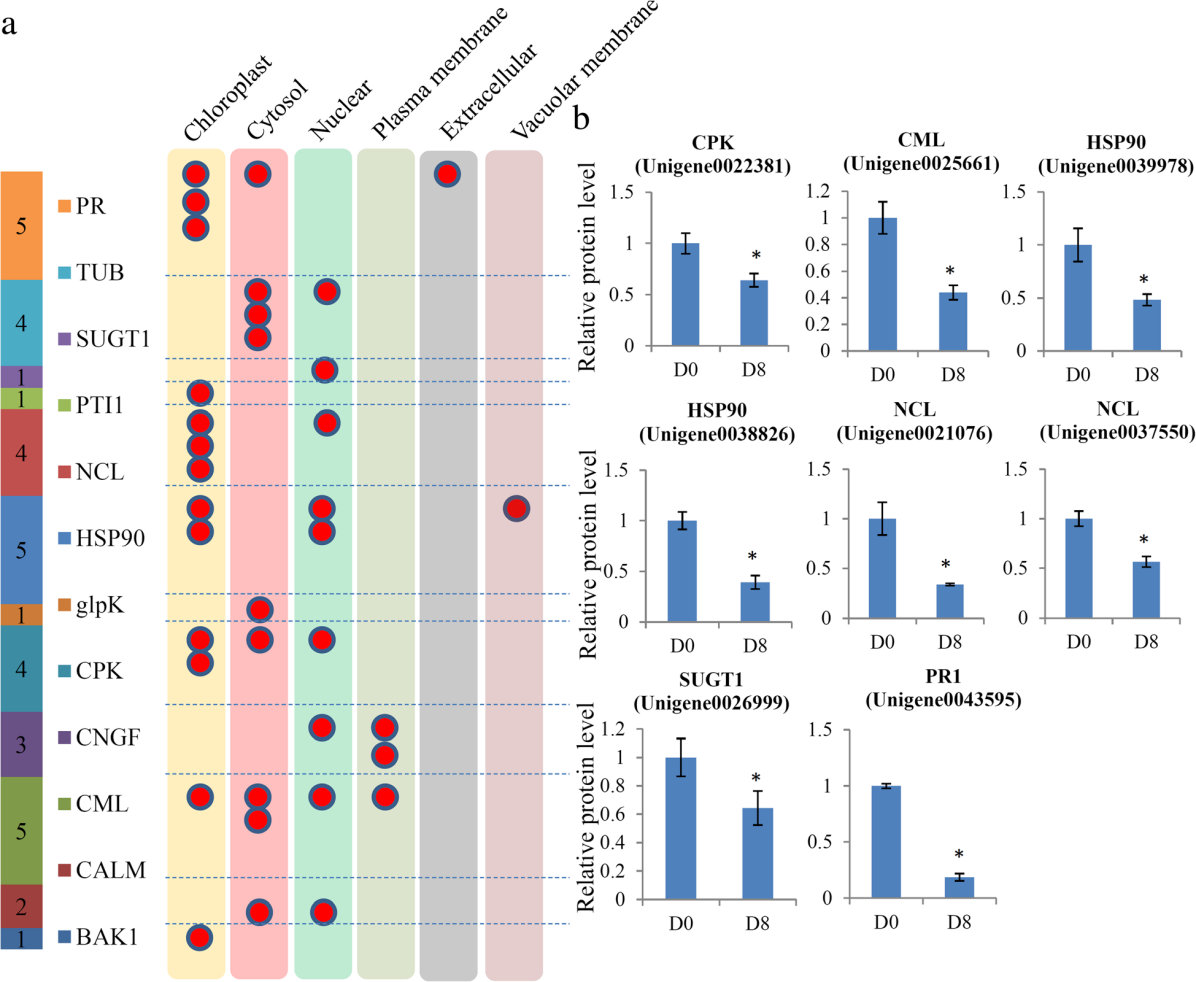
Analysis of the pathogen defense-related proteins during the papaya fruit ripening process. (**a**) The subcellular locations of these pathogen defense-related proteins in papaya. (**b**) Relative expression levels of the proteins related to pathogen defense. Significant differences in expression level were indicated by “*”

We identified a number of pathogen defense-related proteins: five pathogenesis-related (PR) proteins, four tubulin (TUB) family proteins, one suppressor of G2 allele of SKP1 (SUGT) protein, one plant–pathogen interacting (PTI) protein, four nucleolin (NCL) proteins, five heat shock proteins (HSPs), one glycerol kinase (glpK) protein, four calcium-Dependent protein kinase (CPK) proteins, three cyclic nucleotide gated channel (CNGF) proteins, five calcium-binding proteins (CMLs), two calmodulin (CALM) proteins and one brassinosteroid-insensitive 1-associated receptor kinase 1 (BAK1) (Additional file 7). Then, the subcellular locations of these pathogen defense-related proteins were predicted, mostly in chloroplast, cytosol and nucleus. Additionally, two CNGFs and one CML were predicted as plasma membrane located, one PR protein was extracellularly located and one HSP90 protein was located in vacuolar membrane (Fig. 4a).

Among these pathogen defense-related proteins, nine were quantified as DAPs during papaya fruit ripening and, interestingly, most were significantly down-regulated (Fig. 4b).

### Involvement of fatty acid metabolism in the fruit ripening process

**Fig. 5:**
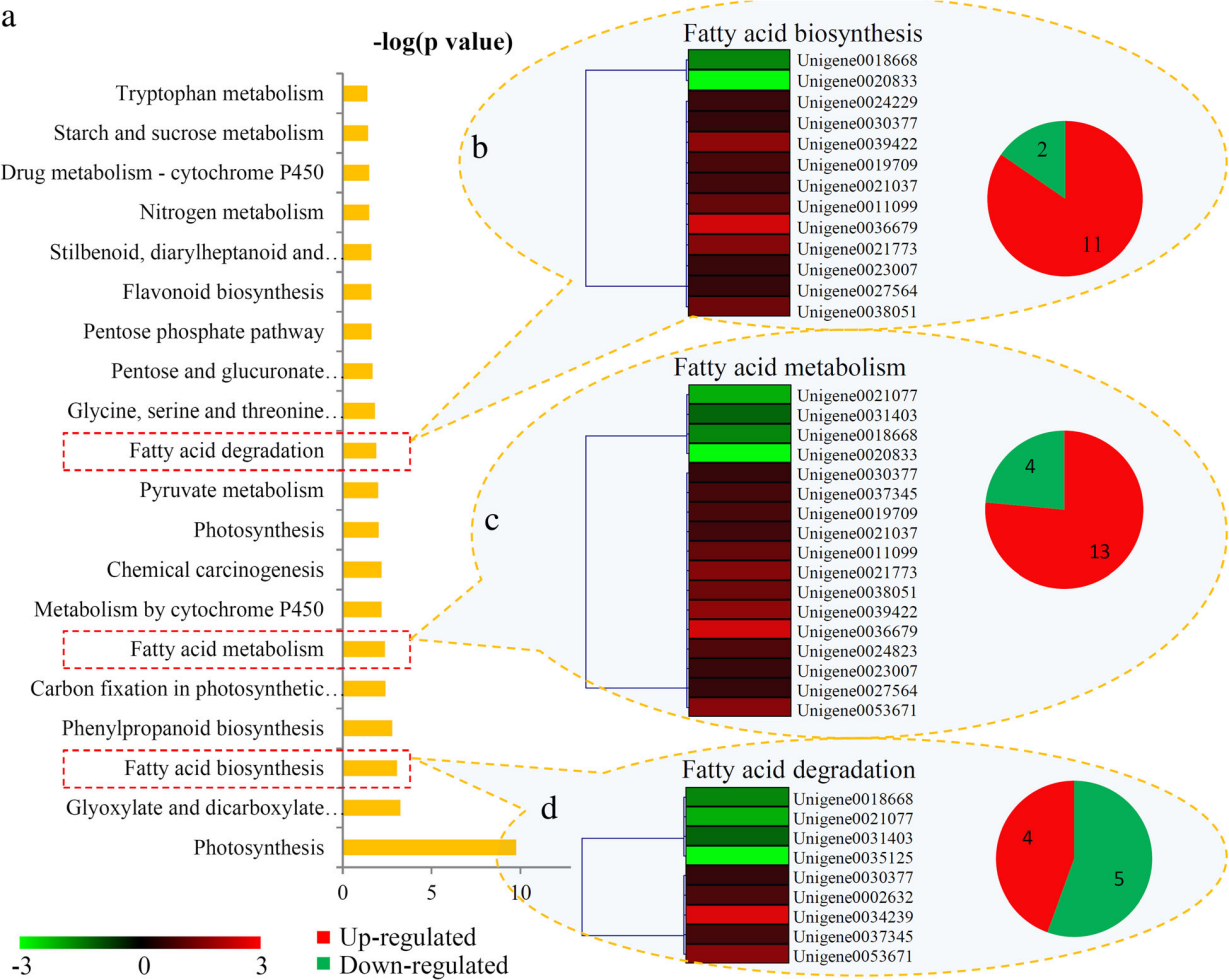
Identification and expression analysis of the KEGG terms related to Fatty acid metabolism. (**a**) KEGG enrichment analysis of DAPs. Differential expression profiling of ‘fatty acid biosynthesis’-(**b**), ‘fatty acid metabolism’-(**c**), and ‘fatty acid degradation’-related genes (**d**) under the fruit ripening process. Red indicated the number of up-regulated genes and green indicated the number of down-regulated genes

Three fatty acid related metabolism pathways were significantly changed during the ripening process of papaya fruit: ‘Fatty acid biosynthesis’, ‘Fatty acid metabolism’ and ‘Fatty acid degradation’ (Fig. 5a). For the ‘Fatty acid biosynthesis’ pathway, two down-regulated and 11 up-regulated proteins were identified (Fig. 5b); for the ‘Fatty acid metabolism’ pathway, four down-regulated and 13 up-regulated (Fig. 5c); and for the ‘Fatty acid degradation’ pathway, four down-regulated and five up-regulated (Fig. 5d). Enzymes involved in fatty acid metabolism are listed in Additional file 8.

**Fig. 6:**
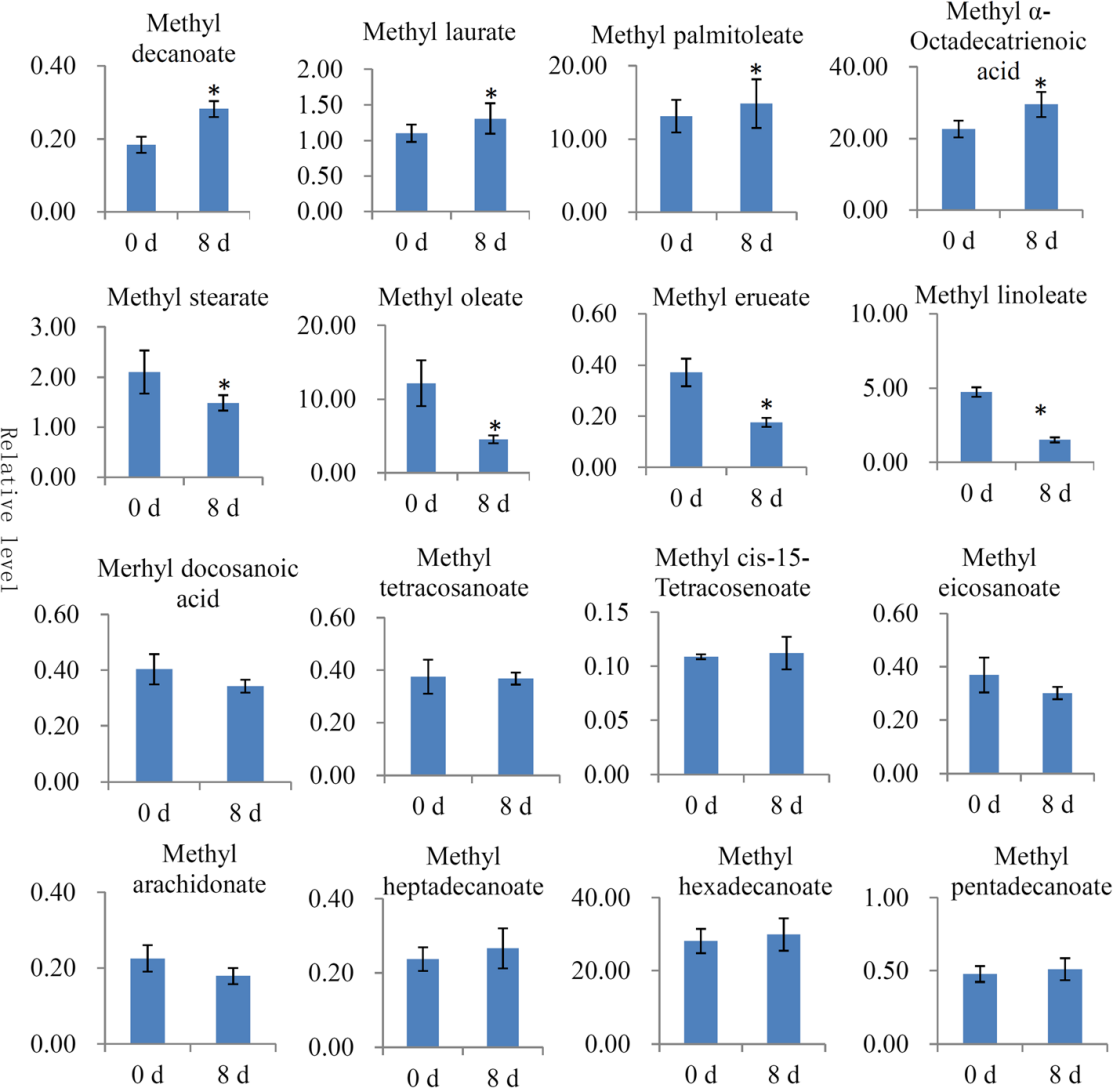
The changes in the contents of various fatty acids during the papaya fruit ripening process. Significant differences in contents were indicated by “*”

Furthermore, the contents of several important fatty acids were determined during the fruit ripening process. In total, 16 kinds of fatty acids were identified and respectively quantified at 0 and 8 d (Fig. 6). Notably, four fatty acids – methyl-decanoate, methyl laurate, methyl palmitoleate and methyl α-octadecatrienoic acid – were significantly up-regulated at 8 d compared with 0 d; and four fatty acids – methyl stearate, methyl oleate, methyl erueate and methyl linoleate – were significantly down-regulated. Contents of the remaining eight fatty acids did not significantly change.

### Involvement of cell wall-related proteins in the fruit ripening process

A number of enzymes, including PG, pectinesterase (PE), α-galactosidase (α-GAL), β-GAL, β-xylosidase, PL, expansin (EXP), xyloglucan-endotransglycosylase (XET) and endoglucanase (EG), are known to be involved in cell wall metabolism [32]. In our study, three PGs, six PEs, two α-GALs, one β-GAL, two EXPs, two XETs and two EGs were identified as DAPs. The expression patterns of the cell wall metabolism-related proteins were thus analyzed during the ripening process: all PGs, α-GALs and most PEs (except for Unigene0023139) were significantly down-regulated; and all β-GAL, EXPs, EGs and one EXT (Unigene0032975) were significantly up-regulated (Additional file 9).

### Verification of the changes in fruit ripening-related proteins using PRM

**Fig. 7:**
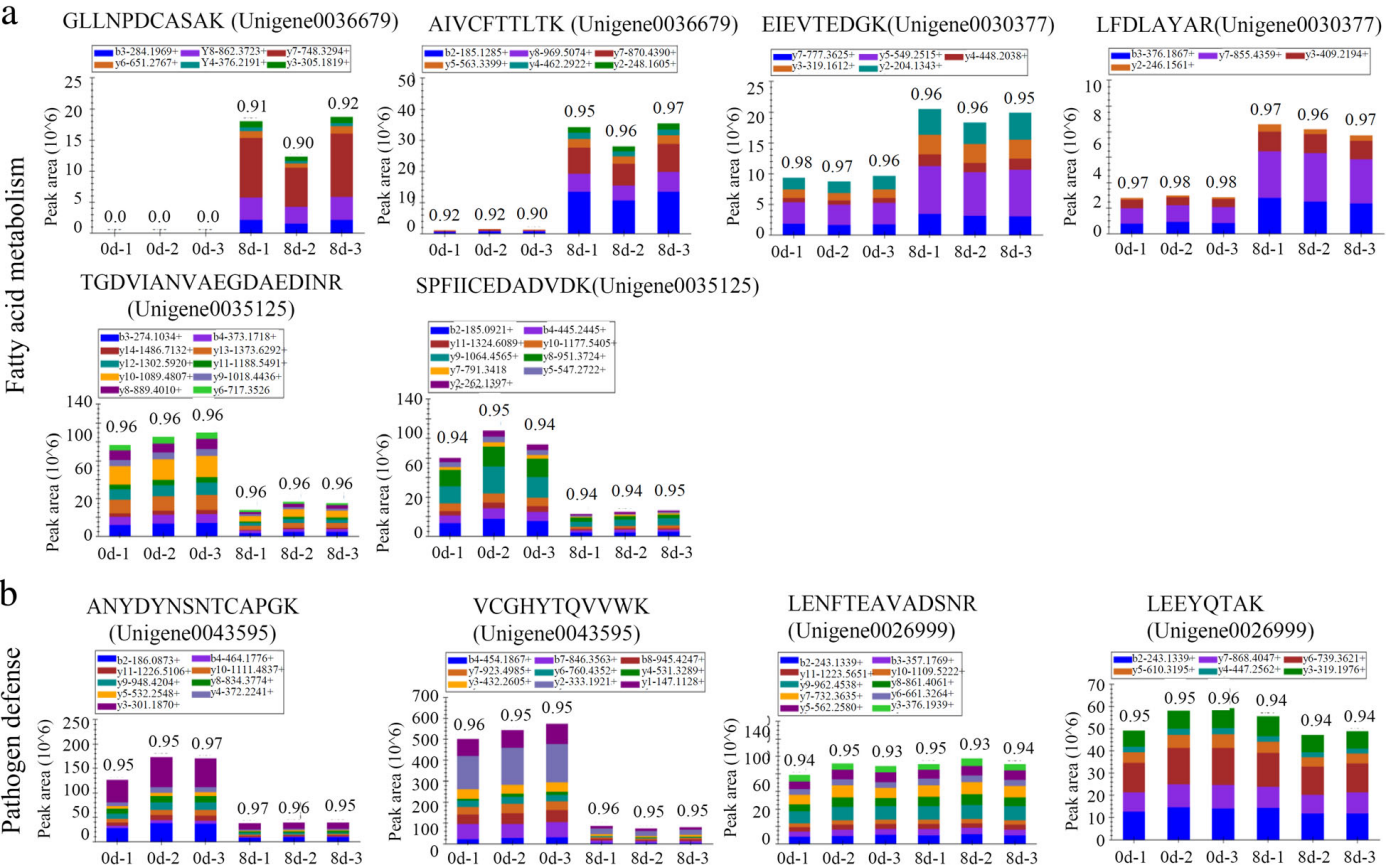
Verification of the changes in fatty acid- and pathogen defense-related proteins using PRM. Five representative proteins, including three proteins involved in fatty acid metabolism (**a**), two pathogen defense-related proteins (**b**), were randomly selected for PRM verification. For each protein, the abundances of two peptides were determined

**Fig. 8:**
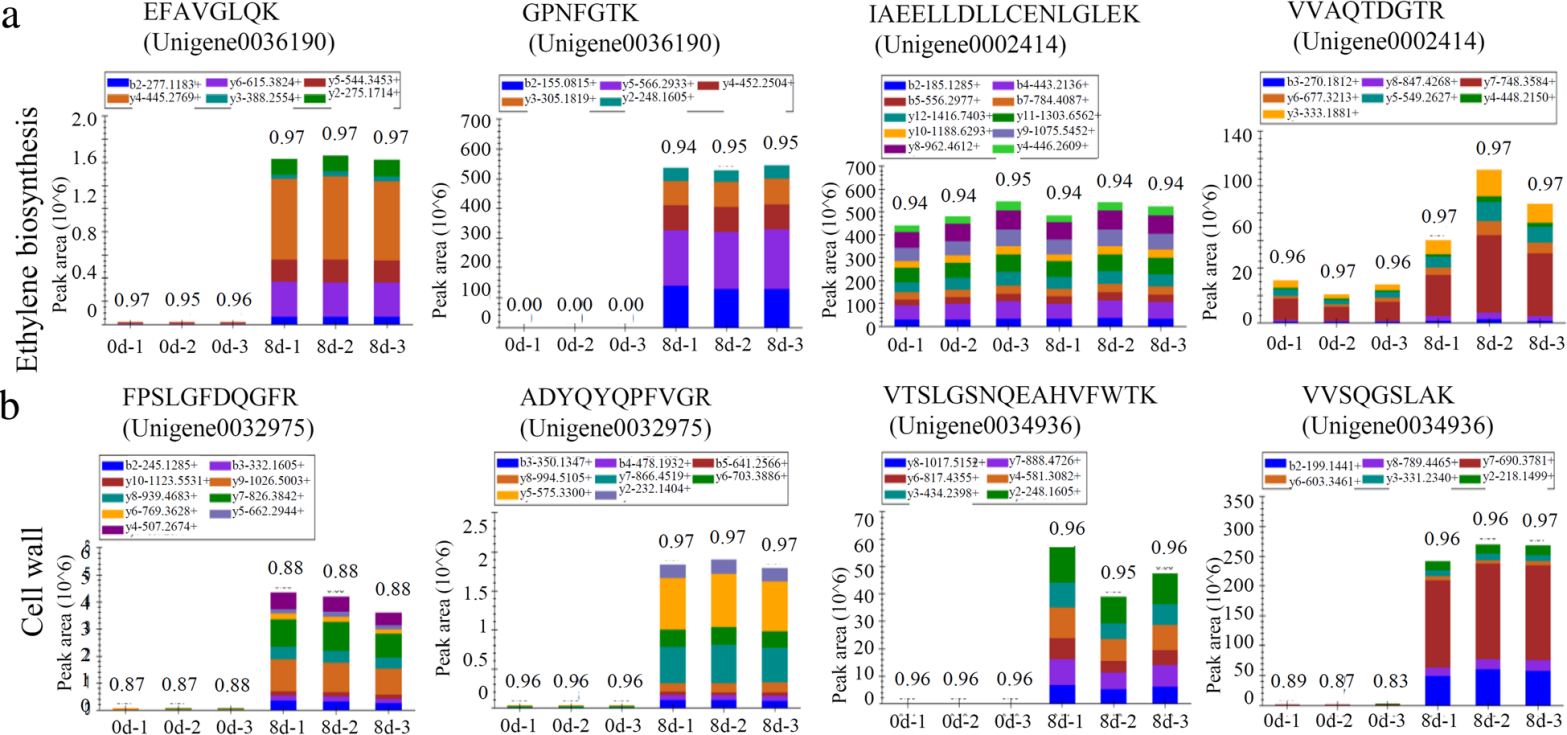
Verification of the changes in ethylene biosynthesis- and cell wall-related proteins using PRM. Nine representative proteins, including two proteins involved in ethylene biosynthesis (**a**), and two cell wall-related proteins (**b**), were randomly selected for PRM verification. For each protein, the abundances of two peptides were determined

Because fruit ripening is extremely complex, it is difficult to identify a single sensitive biomarker. Instead, the identification of a panel of candidate enzymes that reflect the process of fruit ripening would offer a better understanding of this complex process. To validate the differential expression of several key enzymes involved in papaya fruit ripening, PRM was applied. In total, 16 key proteins, including five fatty acid metabolism-related, two pathogen defense-related, two ethylene biosynthesis-related and seven cell wall biosynthesis-related proteins, were selected for PRM verification (Additional file 10). The relative abundances of several key proteins from different sample groups are presented in Figs. 7 and 8. The trend of these DAPs determined by PRM was consistent with our TMT-label quantification results.

## Discussion

Fruit ripening involves a process of several modifications, including starch–sugar conversion, cell wall degradation, flavor substance and pigment biosynthesis, and metabolic rearrangement [32–34]. As climacteric fruit, softening of papaya is an important feature that determines its marketing value [35]. Increasing numbers of studies have focused on molecular and biochemical mechanisms underlying the regulation of climacteric fruit ripening. In our study, comprehensive analysis was carried out to identify the DAPs involved in the ripening process.

Several proteomic analyses of papaya fruit have been performed using the 2DE method. For example, proteomic analysis of PMeV-infected papaya identified a total of 486 reproducible spots, among which calreticulin and the proteasome subunits 20S and RPT5a were upregulated during infection [36]. A differential analysis of the proteomes of climacteric and non-climacteric papaya fruit revealed 27 proteins with high differences in levels during ripening [37]. Comparative proteomic analysis of somatic embryo identified 76 spots, suggesting a role of polyethylene glycol in somatic embryo development in papaya [26]. We identified a total of 3220 proteins and 2818 proteins were quantified, which is more than in previously published proteomes of papaya fruit [25, 37]. Embracing comprehensive information allowed us to explore new proteins/genes that were potentially involved, directly and indirectly, in the ripening process.

A sharp increase in ethylene production is considered a marked feature of climacteric fruits at the initial stage of ripening [38]. In higher plants, the important roles of two enzymes, ACC synthase and ACC oxidase, in ethylene biosynthesis have been well-studied [39]. In papaya, two different ACO genes were identified as participating in papaya fruit ripening [40]. In the up-regulated protein list, the most remarkable were two ACC oxidases, up-regulated during the ripening process by 7.6 and 1.8 fold, respectively (Additional file 1). A similar result was also reported in a 2DE-DIGE analysis [37]. Our data confirmed the critical role of ethylene in papaya fruit ripening.

The ripening of fleshy fruit is associated with changes in susceptibility to pathogen infection and resistance to pathogen infection significantly decreases during ripening [41]. Pathogen-related proteins (PRs) play a role in multiple functions, particular in defense against pathogens [42]. In papaya fruit, eight pathogen-related factors were significantly reduced and only one significantly induced (Fig. 4b), indicating that the disease resistance of papaya fruit might decrease during ripening.

The metabolic shifts throughout ripening cause alterations in primary and secondary metabolites, such as amino acids, fatty acids, sugars, polyphenols and cinnamic acids [43]. Our KEGG enrichment analysis revealed that various metabolic pathways were significantly changed during ripening, suggesting a close relationship between metabolism and fruit ripening of papaya. Flavonoids, the ubiquitous fruit secondary metabolites, play important roles in coloring, abiotic stress defense and other biological functions [44]. In date palm, proteins involved in anthocyanin biosynthesis were up-regulated particularly from the onset of ripening and during ripening [45]. Similarly, during the ripening of papaya, several flavonoid biosynthesis-related proteins were up-regulated: cinnamate-4-hydroxylase, coumaroyl 3-hydroxylase and flavanone-3-hydroxylase (Fig. 5 and Additional file 1). Increases in flavonoids may provide raw materials for pigment biosynthesis, accelerating the color variation of papaya fruit [46].

Interestingly, three fatty acid metabolic pathways were highlighted by our KEGG enrichment analysis, and a number of fatty acid metabolism-related enzymes were also identified as DAPs (Fig. 5). Studies on the changes in fatty acid composition during fruit development and ripening have been performed for various fruit plants. For example, the levels of ω-6 and ω-3 fatty acids significantly changed during mango fruit development and ripening [47]. Ripening raises the content of polyunsaturated fatty acids in tomato fruit [48]. In our study, assessment and profiling assessed the variation of fatty acids during the ripening process of papaya fruit (Fig. 6). Among 16 quantified fatty acids, eight showed significant changes during the ripening process (Fig. 6). In tomato fruit, ripening increases the contents of unsaturated fatty acids [48]. In papaya, two unsaturated fatty acids, methyl palmitoleate and methyl α-octadecatrienoic acid, were significantly up-regulated during ripening. Increases in unsaturated fatty acids might be associated with papaya fruit volatile formation [49].

## Conclusions

In summary, a total of 3220 proteins were detected, 2828 of these were quantified. Among the DAPs, most pathogen-related proteins were down-regulated during the ripening process. Moreover, flavonoid and fatty acid metabolisms were greatly changed during ripening; and unsaturated fatty acids may be associated with papaya fruit volatile formation. Our data may give an intrinsic explanation of the variations in metabolism and offer important information about how to expend the shelf life of postharvest papaya fruit.

## Additional files

Additional file 1: **Table S1**. The detail information of the identified proteins. (PDF 316 kb)

Additional file 2: **Figure S1.** Variations in protein abundances during the papaya fruit ripening. (XLSX 747 kb)

Additional file 3: **Table S2.** The detail information of the DAPs during fruit ripening. (XLSX 118 kb)

Additional file 4: **Table S3**. The scores for the subcellular location of each protein. (XLSX 141 kb)

Additional file 5: **Figure S2.** KEGG enrichment analysis of the DAPs during the ripening process of papaya fruits. (PDF 175 kb)

Additional file 6: **Figure S3.** Protein domain enrichment analysis of the DAPs during the ripening process of papaya fruits. (PDF 308 kb)

Additional file 7: **Table S4.** The detail information of the pathogen defense-related proteins. (XLSX 17 kb)

Additional file 8: **Table S5.** The information of the fatty acid metabolism-related proteins. (XLSX 14 kb)

Additional file 9: **Table S6.** The information of the proteins involved in the cell-related process. (XLSX 10 kb)

Additional file 10: **Table S7.** The PRM results of 16 key proteins. (XLSX 13 kb)

## Data Availability

The datasets generated and analysed during the current study are available in the ProteomeXchange Consortium by PRIDE partner repository program under identifier PXD008871.

## Abbreviations

1-MCP: 1-methylcyclopropene
2-DE: two-dimensional gel electrophoresis
ABA: abscisic acid
ACO: ACC oxidase
BAK1: brassinosteroid-insensitive 1-associated receptor kinase 1
CALM: calmodulin
CML: calcium-binding proteins
CNGF: cyclic nucleotide gated channel
CPK: calcium-Dependent protein kinase
DAP: differential accumulated protein
EG: endoglucanase
EXP: expansin
FA: formic acid
FAA: formalin acetic acid
glpK: glycerol kinase
GO: Gene ontology
HPLC: high-performance liquid chromatography
HSP: heat shock protein
JA: jasmonic acid
KEGG: Kyoto Encyclopedia of Genes and Genomes
LC–MS/MS: liquid chromatography-mass spectrometer
NCL: nucleolin
PE: pectinesterase
PR: pathogenesis-related
PRM: Parallel Reaction Monitoring
PTI: plant–pathogen interacting
SUGT: suppressor of G2 allele of SKP1
TEAB: triethylammonium bicarbonate
TUB: tubulin
XET: xyloglucan-endotransglycosylase
α-GAL: α-galactosidase
β-GAL: β-galactosidase
