# Supplementary material for: Comparative proteomic analysis provides novel insights into the regulation mechanism underlying papaya (Carica papaya L.) exocarp during fruit ripening process
Source: BMC Plant Biol. 2019 Jun 6;19:238. doi: 10.1186/s12870-019-1845-4 (PMC6554998; doi:10.1186/s12870-019-1845-4)
Supplement: Supplementary file 1 — Table S1. The detail information of the identified proteins. (PDF 316 kb) [file 12870_2019_1845_MOESM1_ESM.pdf]

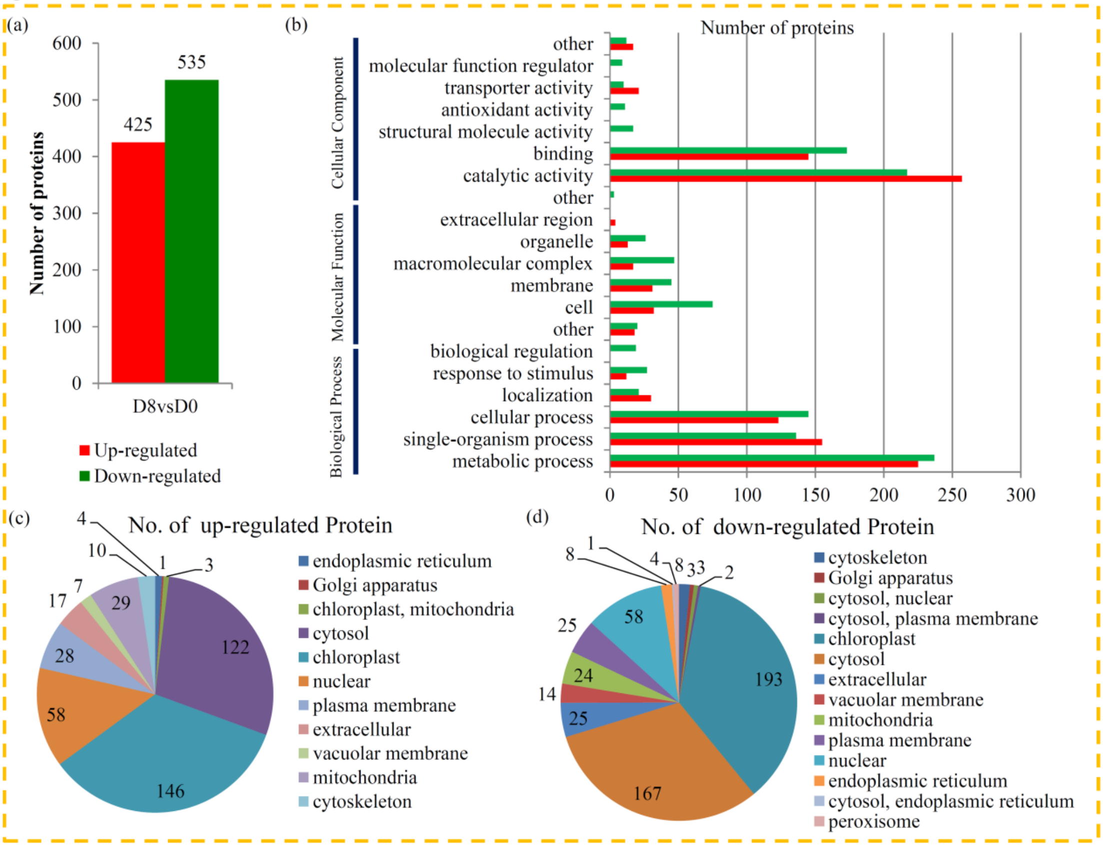

Figure S1 **Variations in protein abundances during the papaya fruit ripening.** (a) The numbers of up- and down-regulated proteins in the fruits at D8 compared with the control fruits (D0). (b) GO analysis of the up- and down-regulated proteins. All proteins were classified by GO terms based on their cellular component, molecular function, and biological process. Subcellular localization of up-regulated proteins (c) and down-regulated proteins (d).
