# Supplementary figures and images for: Comparative proteomic analysis provides novel insights into the regulation mechanism underlying papaya (Carica papaya L.) exocarp during fruit ripening process
Source: BMC Plant Biol. 2019 Jun 6;19:238. doi: 10.1186/s12870-019-1845-4 (PMC6554998; doi:10.1186/s12870-019-1845-4)

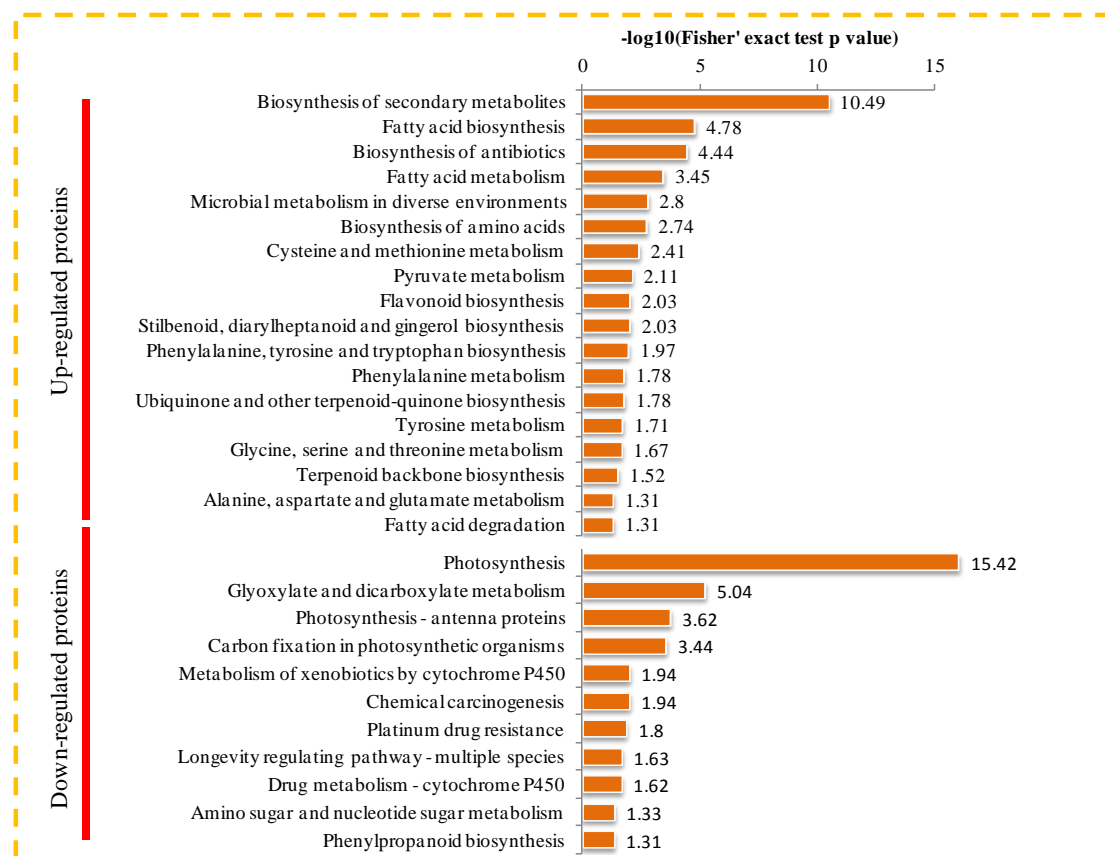

Figure S2 KEGG enrichment analysis of the DAPs during the ripening process of papaya fruit.

Supplement: Supplementary file 5 — Figure S2. KEGG enrichment analysis of the DAPs during the ripening process of papaya fruits. (PDF 175 kb) [file 12870_2019_1845_MOESM5_ESM.pdf]
